# Angular vessels as a new vascular pedicle of an island nasal chondromucosal flap: Anatomical study and clinical application

**DOI:** 10.3892/etm.2012.860

**Published:** 2012-12-18

**Authors:** DIANJU HOU, LIN FANG, ZHENMIN ZHAO, CHUANDE ZHOU, MINGYONG YANG

**Affiliations:** Micro-Invasive Plastic Surgery Center, Plastic Surgery Hospital, Chinese Academy of Medical Sciences and Chinese Peking Union Medical College, Beijing 100144, P.R. China

**Keywords:** eyelid reconstruction, chondromucosal flap, angular vessels

## Abstract

Successful eyelid reconstructions have been reported when using an axial nasal chondromucosal flap based on the dorsal nasal artery. The present study aimed to present a detailed anatomical description of the blood supply of the lateral nasal region and the angular artery, in order to propose the angular vessels as a new vascular pedicle for the island nasal chondromucosal flap. A total of 11 cadavers (22 hemi-faces) were examined. Observations with regard to the origin, course and distribution patterns of the angular artery were recorded. Based on the anatomical study findings, the angular vessels were proposed as a vascular source for the island nasal chondromucosal flap. Observations with regard to the varying origins of the angular artery were categorized into four types. The course of the angular artery along the nasojugal fold was constant. The angular artery branched off into the upper two-thirds of the lateral nasal region and anastomosed with the other vascular branches on the nasal dorsum. Clinically, reconstruction of a full-thickness defect of the lower eyelid was successfully performed by using this composite flap based on the angular vessels and an adjacent orbicularis oculi myocutaneous flap. Satisfactory esthetic outcomes were obtained for the donor and recipient sites. The angular artery is a good vascular source for an island nasal chondromucosal flap. The flap may be created safely and successfully in clinic. Island nasal chondromucosal flaps and nasolabial groove skin flaps based on the angular vessels may be designed simultaneously for use on full-thickness defects of the eyelid.

## Introduction

In the reconstruction of large full-thickness eyelid defects, it is difficult to find a suitable tissue for functional repair. Scuderi *et al* first reported the use of an axial nasal chondromucosal flap for reconstruction of the tarsoconjunctival plane in full-thickness eyelid defects (1–4). The technique is associated with the use of a local skin flap or with a skin graft for skin repair. The axial chondromucosal flap receives its blood supply from the dorsal nasal artery. The question of whether the angular artery may be used as a pedicle of the island nasal chondromucosal flap remains to be clarified.

Currently, there is a lack of detailed description on the angular artery, even where the retroangular artery flap has been widely used. Therefore, in the present study, a detailed analysis was performed on the angular artery. The different origins, course, distributions and surrounding relationships of the angular arteries were investigated. Utilizing the findings of this anatomical study, an island nasal chondromucosal flap based on the angular artery was used for clinical cases of full-thickness lower eyelid defects, in association with an orbicularis oculi myocutaneous flap for skin repair.

## Materials and methods

### Cadavers

Once institutional approval from the Chinese Peking Union Medical College was obtained to perform the present study using cadavers, 11 Chinese adult cadavers, including 7 males and 4 females (22 hemi-faces), were dissected to investigate the relationship between the angular artery and other anatomic structures of the nose.

### Anatomical study in cadavers

The present study was conducted in accordance with the declaration of Helsinki. Written informed consent had been previously obtained from all participants. Prior to dissection, the cadavers were injected with a red latex solution into the bilateral common carotid arteries and two of the cadavers were injected bilaterally with a blue latex solution into the facial veins. The dissections were carried out from superficial to deep levels, and the vessels and nerves were preserved as they were revealed. The vascular and nervous anatomy of the nasal dorsum and paranasal region was revealed under ×3.5 loupe magnification.

### Clinical application

The anatomical results identifed in the present study were then applied to clinical research.

### Surgical technique

Flap elevation involves simple surgical steps and can be performed under local anesthesia. In this study, the surgical technique was applied to the clinical cases.

The flap based on the angular vessels, including the cranial portion of the upper lateral cartilage and the corresponding nasal mucosa, was harvested depending on the size of the defect to be repaired. The cartilage harvest range utilized is 15–18×5–8 mm. The mucosal portion of the flap can be extended, depending on the reconstructive need.

Following skin incision of ∼20–30 mm along the nasojugal fold from the ala nasi up to the direction of inner canthus, the subcutaneous dissection was extended superiorly to the inner canthus and glabellum, and inferiorly to the lower margin of the upper lateral cartilage. The angular artery was identified using a preoperative Doppler probe and an intraoperative manual palpation. During surgery, pressure was applied manually on the artery at the lower border of the nasal alar groove to ensure blood flow of the angular artery from the cephalic to the caudal region. The angular artery and vein are close together, therefore, their location in the pedicle of the flap was confirmed prior to attempting dissection downwards. The pedicle includes the angular vessels, a piece of muscle around them, and the infratrochlear nerve. An incision was made through the upper lateral cartilage, including the perichondrium and the nasal mucosa at the distal end of the flap. From both ends of that incision, two vertical cuts were made at a level where a part of the upper lateral cartilage was to be included, based on the width of the defect to be repaired. The flap was then raised (Fig. 8). Formation of the neurovascular pedicle should be dissected in consideration of the rotation arc from the donor to the defect site. Under certain conditions, this pedicle may be extended to the medial canthus. A tunnel was dissected in the subcutaneous plane of the infraorbital and medial canthal region. The flap, elevated over the pedicle through careful dissection, was passed through the tunnel and sutured in order to reconstruct the tarsoconjunctival plate of the missing eyelid. Meticulous attention was paid to the graft, and sutures and knots on the conjunctival surface were avoided. The donor site was closed immediately, and a nasal pack was applied for 48 h.

Reconstruction was then performed with a chondro mucosal flap based on the angular vessels and the accompanying nerve, associated with an orbicularis oculi myocutaneous flap for skin repair.

## Results

### External nose vascular supply

**Figure 1.**
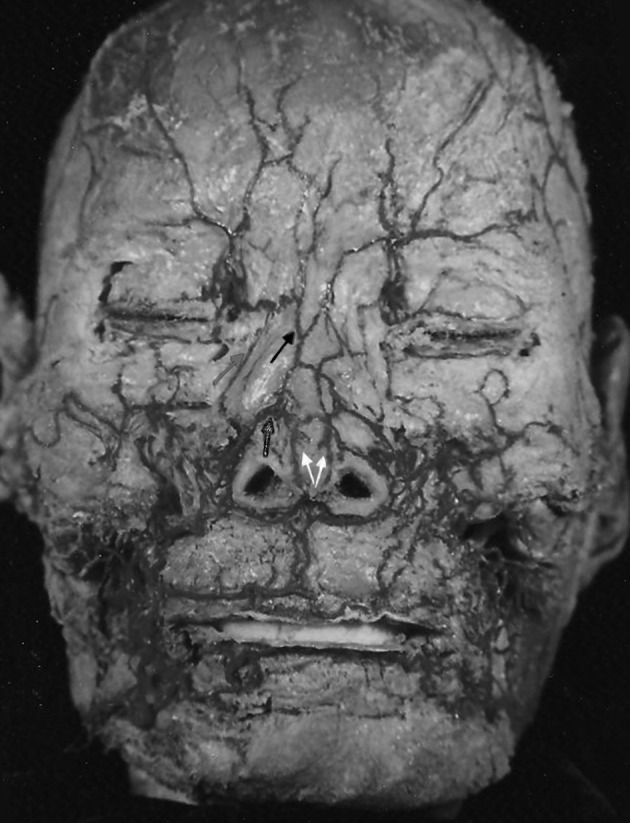
External nose vascular supply. Gray arrow: angular artery. Black arrow: dorsal nasal artery. White arrows: small columellar arteries. Dotted white arrow: lateral nasal artery. Asymmetry of the vascular patterns between the two hemi-noses was encountered.

The external nose received its blood supply from the lateral and dorsal nasal, angular and small columellar arteries. These arteries were structured as branches and anastomoses, forming a vascular network (Fig. 1).

Originating from the ophthalmic artery, the dorsal nasal artery descended along the dorsum, terminated at the alar groove and was anastomosed with the lateral nasal artery.

The lateral nasal artery originated mainly from the facial artery. In certain samples, the facial artery did not terminate at the nasal ala, thus the lateral nasal artery originated from the infraorbital artery or the nasoseptal artery from the superior labial artery.

The small columellar artery, a branch of the superior labial artery of the facial artery, ran upward in the columella and parallel to its contralateral partner.

The angular arteries (angled where the upper and lower eyelids meet) traveling along the nasojugal fold (with one end near the alar groove and the other end near the medial canthus) were consistently observed in all specimens. The angular artery branched out into the upper two-thirds of the lateral nasal region and anastomosed with other vascular branches of the nasal dorsum. The artery was involved in the composition of a reliable vascular arcade in the external nasal region.

### Angular artery origin

**Figure 2.**
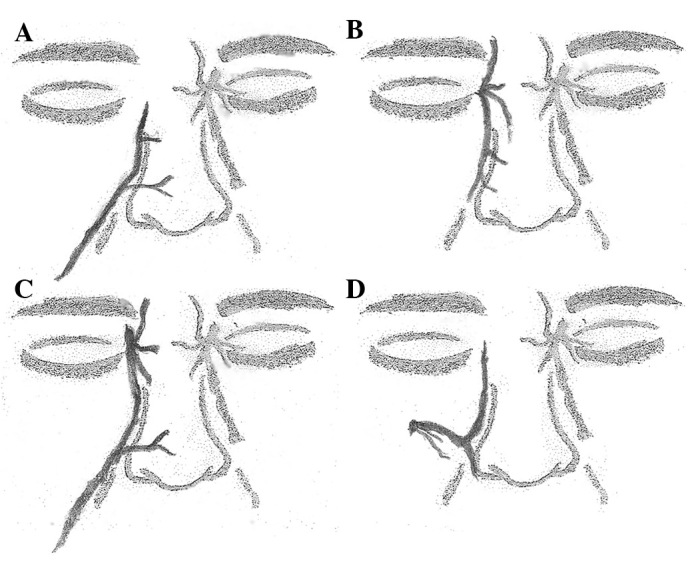
Categorization of the angular arterial origin. Schematic diagram showing the four types of angular artery origin. (A) (TypeI) The angular artery originated from the facial artery. (B) (Type II) The angular artery originated from the ophthalmic artery. (C) (Type III) The angular artery was an anastomosis consisting of the terminal branch of the facial dorsal nasal arteries. (D) (Type IV) The angular artery originated from the infraorbital artery.

**Figure 3.**
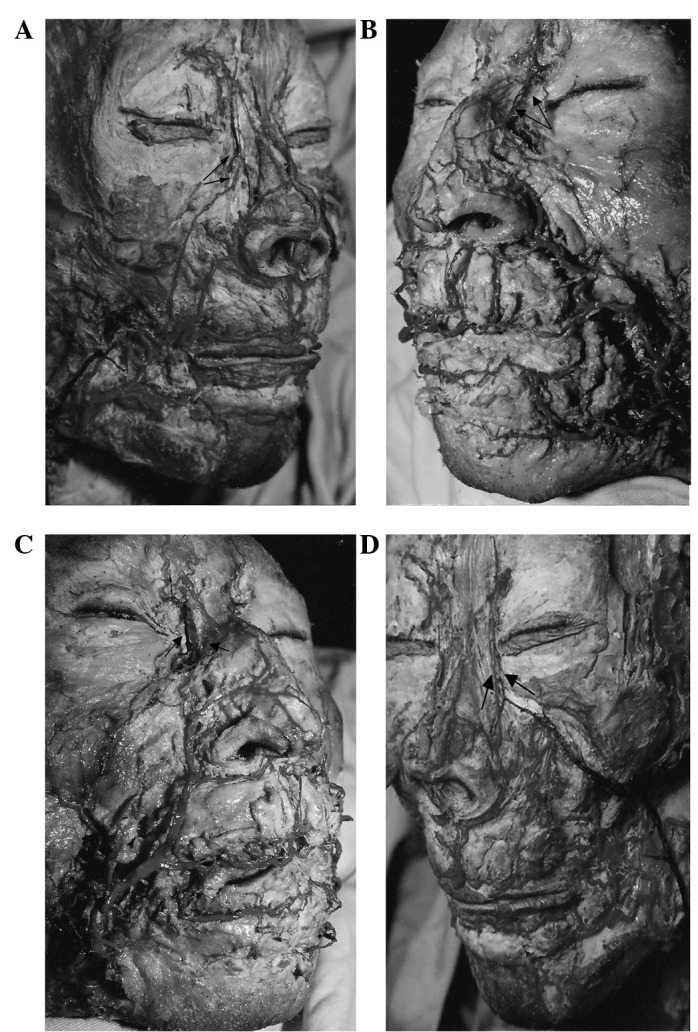
The different angular origins. Black arrows showing the angular vessels. (A) Type I: The angular artery originated from the facial artery. (B) Type II: The angular artery originated from the ophthalmic artery. The artery was superficially embedded in the fibres of the levator labii superioris alaeque nasi and the vein was located at a deeper level with the angular artery at the medial canthus. (C) Type III: The angular artery was an anastomosis consisting of the terminal branch of the facial dorsal nasal arteries. (D) Type IV: The angular artery originated from the infraorbital artery.

**Figure 4.**
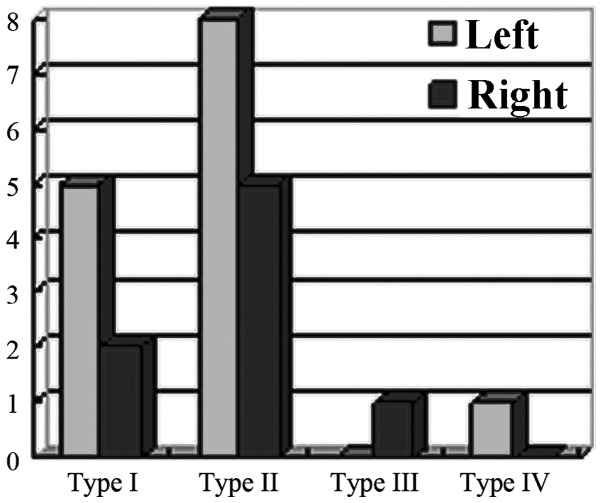
Typing of arterial origin of the angular artery in the cadavers on the left and right side. Assymetry between the two hemi-noses was revealed in the same specimen in 6 cases.

The angular artery was found to originate from various other arteries. Four types of sources were observed in the specimens (Figs. 2 and 3). In Type I examples, the angular arteries originated at the terminal branch of the facial artery. The facial artery branched out as the superior and inferior labial arteries and the lateral nasal artery and then terminated as the angular artery, which ran through the side of the nose and the medial canthus. This type was observed in seven hemi-faces (31.8%, 5 in left hemi-faces, 2 in right; Fig. 3A). In Type II, the angular artery was revealed to originate from the ophthalmic artery. The blood flow of the angular artery was from top to bottom and its terminal branches were in the paranasal region and the nasal dorsum. In this type, 13 hemi-faces of specimens were observed (59.1%, 8 in left hemi-faces, 5 in right; Fig. 3B). In Type III, the angular artery was an anastomosis consisting of the terminal branch of the facial artery and the branch of the dorsal nasal artery. Anastomosis occurred on the surface of the upper lateral cartilage. Type III was observed in 1 hemi-face (4.5%, 1 in right hemi-face; Fig. 3C). In Type IV, the angular artery originated from the infraorbital artery. The infraorbital artery, the terminal branch of the maxillary artery, emerged at the infraorbital foramen and terminated at the angular artery. Type IV was also observed in only 1 hemi-face (4.5%, 1 in left hemi-face; Fig. 3D). Asymmetry of the vascular patterns between the two hemi-noses was encountered in the same specimen in 6 cases (54.5%; Fig. 4).

### Relationship between angular and dorsal nasal arteries

**Figure 5.**
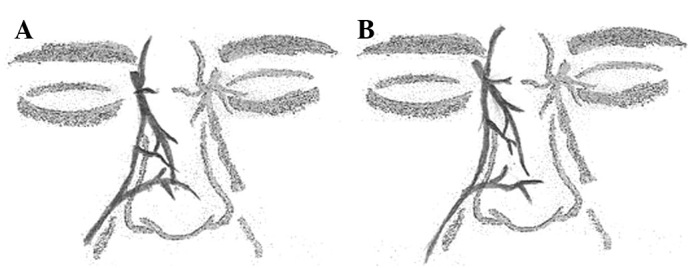
Relationship between the angular artery (Types II and III) and dorsal nasal artery. (A) One-branch type: the angular artery originating from the trunk of the dorsal nasal artery. (B) Multi-branch type: the angular artery originating separately from the ophthalmic artery.

In Types II and III, the angular artery originating from the internal carotid arterial system was observed to have two different special relationships with the dorsal nasal artery (Fig. 5). In the one-branch type, the angular artery shared the trunk of the dorsal nasal artery, while in the multi-branch type, the angular artery originated separately from the ophthalmic artery. Its terminal branches were anastomosed with the branches of the dorsal or lateral nasal artery.

### Course of the angular vessels and surrounding structures

The venous drainage of the external nose had similarly named veins that accompanied the arteries. The blood drained via the facial veins or the pterygoid plexus, specifically via the ophthalmic veins into the cavernous sinus.

**Figure 6.**
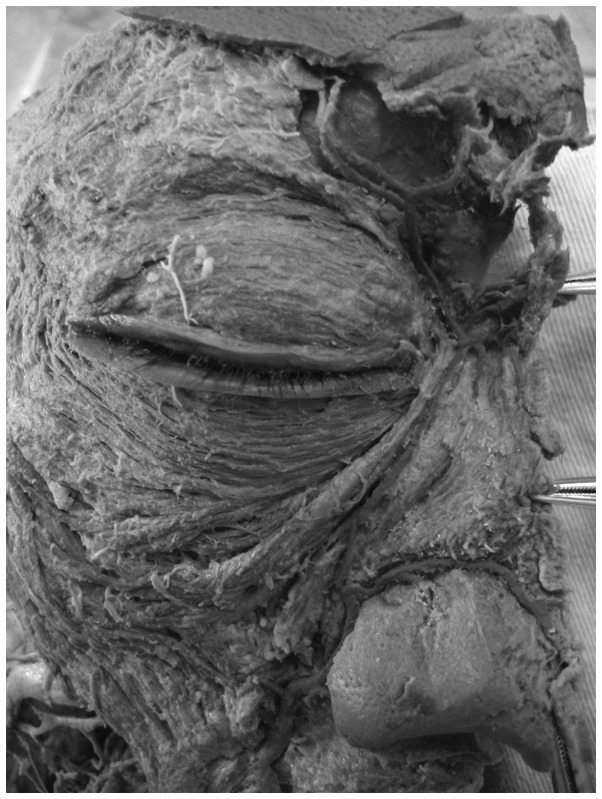
Course of the angular artery. The artery superficially embedded in the fibres of the levator labii superioris alaeque nasi.

The accompanying vein of the angular artery was also observed in specimens of the present study. The angular artery and its vein were located together. The artery was superficially embedded in the fibres of the levator labii superioris alaeque nasi and the vein was located at a deeper level with the angular artery at the medial canthus. The angular vein was medial to the angular artery above the medial canthus and lateral to the angular artery below the medial canthus. At the inferior end of the nasal bone, a vein originating from the surrounding tissues of the upper lateral nasal cartilage was infused into the angular vein, which lay deep in the layer of the levator labii superioris alaeque nasi (Fig. 3B and C and Fig. 6).

Another observation was that the infratrochlear nerve consistently appeared to accompany the angular artery. The nerve gave off branches that reached the nasal lateral region and were distributed into the nasal lateral cartilage and the corresponding mucosa.

### Flap model in cadaver anatomy

**Figure 7.**
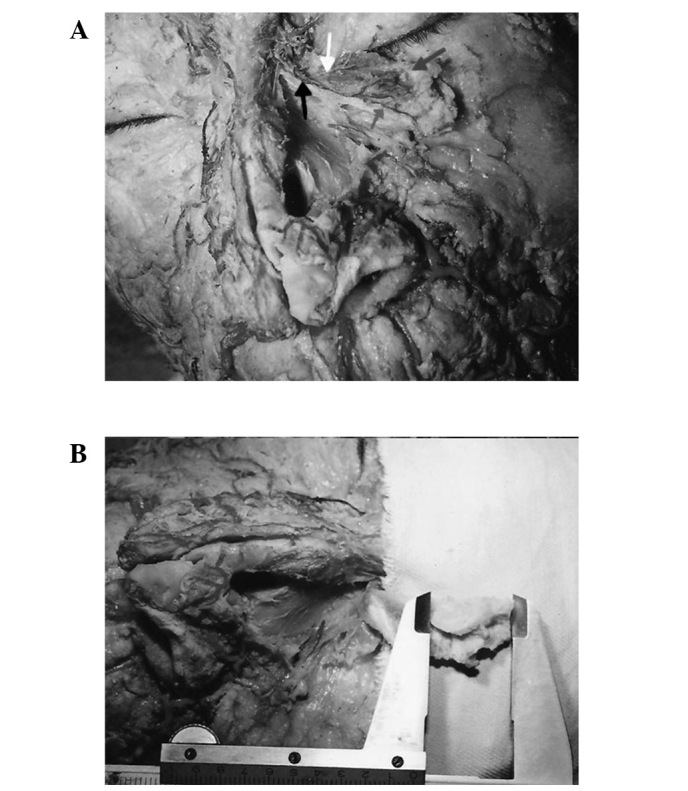
The flap model in the anatomy of a cadaver. (A) Island chondromucosal flap based on the angular vessels and accompanying nerve. The black arrow shows the neurovascular pedicle of this flap. The white arrow shows the branches of the infratrochlear nerve. The gray arrow shows the angular artery and the dotted black arrow shows the angular vein. (B) Cartilaginous and mucosal portion of this flap.

**Figure 8.**
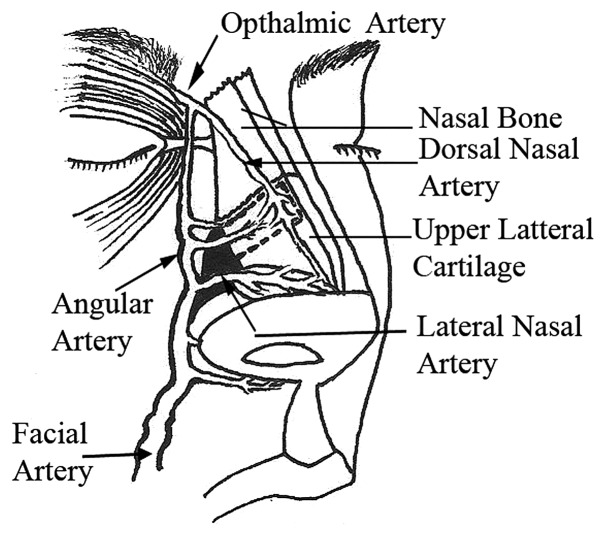
Harvest of the flap. Schematic diagram depicting the harvest of the island chondromucosal flap based on the angular artery. ---- represents the outline of the chondromucosal flap

In the present study, anatomical analysis showed that the angular vessels were constant in course and distribution and branched out into the surrounding tissues of the upper lateral cartilage, even though the angular artery had diverse origins. Therefore, the angular vessels were available for use as a vascular pedicle of an island chondromucosal flap in the lateral side of the nose, adjacent to the nasojugal fold. The blood supply to the flap was derived from the branches of the angular artery, which stretched onto the surface of the upper lateral cartilage. This flap, based on the angular vessels and infratrochlear nerve, was considered able to survive in an eyelid defect reconstruction and provide sensation following a one-stage surgery (Fig. 7).

The anatomical results from the present study were then applied to clinical research.

### Clinical application - a representative case

**Figure 9.**
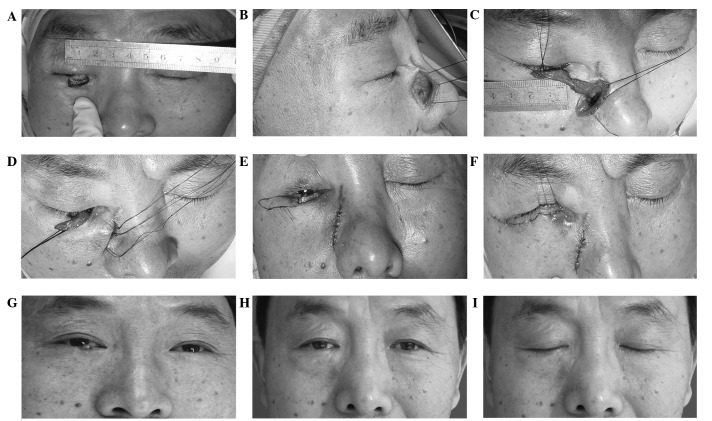
Preoperative, intraoperative, and post-operative views of the patient described in the clinical application of the flap. (A) Eyelid defect following tumor excision. (B) Dissection in the subcutaneous plane. (C) The length of the chondromucosal tissue. (D) The flap passing through the tunnel. (E) Design of the myocutaneous flap. (F) Closure of the wound. (G) Preoperative view. (H) Post-operative view (eye open) 2 years after surgery. (I) Post-operative view (eye closed) 2 years after surgery.

A 52-year-old male patient underwent ablation of a malignant tumor on his right lower eyelid. Following a wide excision of the tumor, there was a resulting 15×10 mm defect of the eyelid. Pathological examination confirmed the absence of malignant cells in the margins of the surgical specimen and therefore reconstruction of the full-thickness eyelid defects was performed during a one-stage surgery with the patient under local anesthesia. An island chondromucosal nasal flap based on the angular artery was used to reconstruct the posterior defects and an adjacent orbicularis oculi myocutaneous flap was used to reconstruct the anterior defects of the eyelid. The post-operative course of the patient was uneventful and the donor site near the nose healed completely. The reconstructed eyelid was not bulky and secondary revisions were not required. A follow-up after two years showed complete recovery of eyelid functions. The patient was extremely satisfied with the esthetic results, which provided a close match to the original quality of the skin and had minimal donor site complications. There was no tumor recurrence, ectropion or entropion, retraction, epiphora or minor conjunctival irritation of the eyelid. No observations of airway obstructions due to a defect in the donor site have been noted (Fig. 9).

## Discussion

Numerous techniques have been utilized for eyelid reconstruction (5–8). An axial chondromucosal flap from the nose based on the dorsal nasal artery was first used for reconstruction of the tarsoconjunctival plane of full-thickness eyelid defects in 1992 (2). The authors reviewed their experience of using the nasal chondromucosal flap for an upper eyelid reconstruction and presented the merits of the technique. Firstly, the chondromucosal flap was observed to be safe, reliable and did not require long-term eye occlusion. The procedure was a one-stage surgery and did not damage the remnant lid (1). Secondly, the presence of vascularized cartilage in the flap warranted the required support of the reconstructed eyelid. Therefore, this reconstruction was anatomically complete and esthetically well-accepted by patients (3). Total reconstructions were also achievable with this technique (9).

Through anatomical study of cadavers, the authors identified that the nasal chondromucosal flap from the lateral side of the nose received its vascular supply not only from the dorsal nasal artery but also from the angular artery. This was involved in the composition of a reliable vascular anastomosis network in the external nasal region.

Although flaps based on the angular artery in an antegrade or retrograde manner are widely used for reconstruction of facial defects (10–14), detailed studies on the angular artery are rare and generally inadequately described in anatomy books and literature.

The angular artery had originally been thought of as the terminal branch of the facial artery (15), however, various final branches of the facial artery have been reported in previous literature. The distribution patterns of the facial artery described in this literature differ significantly from one another. Mitz *et al*(16) stated that the facial artery ended as the angular artery in only 4% of the 50 facial arteries of adult French cadavers. Koh *et al* reported that the facial artery ended as the angular artery in 36.3% of 91 Korean specimens (17). According to Niranjan, the final branch of the facial artery was the angular artery in 68% of 25 British specimens (18). Loukas *et al* examined 284 hemi-faces and reported that the facial artery ended as the angular artery in 51.4% of the cases, the others terminated as the lateral nasal or superior labial artery or as a mere rudimentary branch (19). Nakajima *et al* observed the angular artery in 18 of 25 facial arteries (72%) (20). In the present anatomical study, the facial artery ended as the angular artery in 8 of 22 Asian hemi-faces (36.4%).

Previous studies reported that if the facial artery was absent or poorly developed, the compensation of the blood supply was usually provided by the ophthalmic artery, infraorbital or transverse facial artery in the ipsilateral position or a more developed contralateral facial artery (21,22).

In the present study, a detailed analysis on the origins, courses, distributions and surrounding relationships of the angular artery was performed. The origin of the angular artery was classified into four types. The angular artery is usually an arterial branch rather than a main continuation of the facial artery. Even when the origin of the angular artery was the facial or infraorbital artery, which were allocated as Types I and IV, a reverse blood flow was observed following the blood flow of the facial artery being stopped by the manual application of pressure at the proximal end (23). This procedure confirmed the possibility of safe elevation of an antegrade or retrograde flow-arterialized flap based on the angular artery. When the angular artery originated from the ophthalmic artery (Types II and III), there were two patterns of relationships with the dorsal nasal artery. The trunk of the dorsal nasal artery was shared or the artery originated separately from the ophthalmic artery. The angular artery reportedly anastomosed with the dorsal nasal artery via a thin or thick branch in the region of the medial canthus (24).

Although the angular artery had varying origins, its course and distribution were constant. The angular artery branched out onto the surface of the upper lateral cartilage. The vessel was therefore available for use as a pedicle of the nasal chondromucosal flap. Its accompanying vein was located at a deeper level with the artery. A small vein draining the surrounding tissues of the upper lateral cartilage coursed beneath the levator labii superioris alaeque nasi and was infused into the angular vein at the inferior end of the nasal bone. The infratrochlear nerve consistently appeared to accompany the angular artery. Thus, the pedicle of the island nasal chondromucosal flap includes the angular artery, angular vein, and infratrochlear nerve. To preserve the vein in the pedicle, a strip of muscle fiber from the levator superioris alaeque nasi should be included in the pedicle.

Based on the anatomical findings, the present study suggests the utilization of island nasal chondromucosal flap based on the angular artery for repairing the posterior lamella of the eyelids. In the present clinical case, an island nasal chondromucosal flap based on the angular vessels and the infratrochlear nerve, together with an orbicularis oculi myocutaneous flap, were used successfully to reconstruct full-thickness defects. Excellent results were achieved and nasal distortion or abnormal scarring did not occur.

A flap based on the angular vessels has the same merits as a flap based on the dorsal nasal artery. In addition, the pedicle of the flap is closer to the incision, therefore the pedicle elevation and rotation of the flap are easier. The design of the pedicle does not damage the lateral nasal region, the nasal dorsum or the thin soft tissues on the surface of the nasal bone, allowing the figure of the nose to be maintained. Depending on the reconstruction required, the island nasal chondromucosal flap and a nasolabial groove skin flap based on the angular vessels may be used together during a one-stage surgery.

In conclusion, angular artery is a good vascular source for an island nasal chondromucosal flap. The flap is safe and reliable. An island nasal chondromucosal flap and nasolabial groove skin flap based on the angular vessels may be designed simultaneously for use on full-thickness eyelid defects.
